# Depression Levels among Mothers of Children with Leukemia 

**Published:** 2014-07-20

**Authors:** G Kholasehzadeh, SM Shiryazdi, H Neamatzadeh, N Ahmadi

**Affiliations:** 1 Department of Psychiatry, Shahid Sadoughi University of Medical Sciences and Health Services, Yazd, Iran.; 2 Department of General Surgery, Shahid Sadoughi University of Medical Sciences and Health Services, Yazd, Iran.; 3 Department of Pediatric Hematology, Oncology and Genetic Research Center, Shahid Sadoughi University of Medical Sciences and Health Services, Yazd, Iran; 4 Psychiatry and Psychology Research Centre, Tehran University of Medical Sciences, Tehran, Iran.

**Keywords:** Depression, leukemia, Beck Depression Inventory

## Abstract

**Background:**

The aim of the study was to evaluate the depression levels in mothers of children with leukemia.

**Materials and Methods:**

This single centred, cross-sectional study was conducted among mothers of children with leukemia at the Hematology and Oncology research center, Baghaie-Pour clinic in Yazd City during February through December, 2013. The study sample included 58 mothers with 1-12 year old children with the diagnosis or treated at the Shahid Sadoughi hospital. Socio-demographic characteristics were gathered using a socio-demographic form and Beck Depression Inventory (BDI) was applied to all mothers to assess symptoms of depression. All variables that could potentially impact dependent outcome measures of the BDI were analyzed. These factors were mothers' age, mothers' education, and socioeconomic status of the family, gender of child.

**Results:**

The analysis revealed that mothers of children leukemia had a severe level of depression (p=0.01). 41 participants (70.6%) indicated their current depressive symptoms as being in the severe range, 12 participants (20.6%) in the moderate range, and 5 participants (8.6%) in the mild range or no depression. There was an inverse correlation between the educational level of the mothers and the heads of household, their occupational status, their marital status, their socio-economic condition and depression (p≤0.05). The depression scores of the mothers of patients were higher than those of the controls.

**Conclusion:**

Depression ideation is common among mothers of children with leukemia. There are strong associations with socio-economic condition and high depression levels.

## Introduction

Each year approximately 13,000 children under the age of 20-years are diagnosed with cancer in the U.S. (1). Although survival rates for childhood cancer have increased substantially since the 1970s, approximately 2,200 children die from cancer each year, making the threat of death very real for children and their families (2). The diagnosis and treatment of childhood cancer present numerous challenges and sources of stress for children and their parents (3). Blood malignancies are currently the leading cause of death by cancer in children under the age of 15 in the US. While the number of childhood cancer survivors continues to grow, psychological research on this population has lagged (2,3). 

It has been long recognized that in the early months after diagnosis, the parents of children with cancer often suffer a variety of psychosocial symptoms (4). Results from longitudinal studies show that despite the fact that many parents adapt well to the child’s cancer diagnosis, there is still a significant number of parents who struggle with mental fatigue, anxiety and symptoms of post traumatic stress after treatment of the child’s cancer illness (5). The course and outcome of most childhood illnesses are strongly influenced by both family structure and function. 

 Parents are responsible for the treatment of the most pediatric illnesses. Many family variables are associated with health outcomes across a broad range of chronic illnesses. A large body of research has demonstrated that families have a powerful influence on physical health, including morbidity and mortality (6,7),

As a primary care provider mother’s responsibility increases substantially starting a vicious cycle of anxiety and socio-economic uncertainty leading her to depression much more than the father (8). The available data supports that mothers of children with cancer represent a group prone to high levels of emotional distress, and that the period following their child’s diagnosis and the initiation of treatment may be predominantly stressful and disturbing leading them to depression. Such mothers have difficulty in taking care of themselves, their household and especially their sick children. Many parents continue to suffer from clinical levels of distress, even after five years off treatment of their child.

The aim of this study was to evaluate the depression levels among mothers of children with leukemia.

## Materials and Methods

The study was conducted with 58 mothers of children with blood malignancies who were seen at the Baghaie-Pour Clinic at the Shahid Sadoughi University Hospital, in Yazd between February through December 2013. Study participants were the mothers of children under 12 years old, who had a diagnosis of leukemia for at least four months. Mothers were provided with information and invited to participate in the study. The study was conducted during routine clinic visits in which mothers accompanied their children.

**Data collection**

One to one meeting were held with the mothers in the university hospital. Mothers, children demographics information form and Beck Depression scales was administrated at the same time. The questionnaire consisted of different items that aimed to describe the socio-demographic profile of participants: age and educational level of mother, marital status, family income, current occupation (paid or not), religion (practicing or not), number of children.

**Beck Depression Inventory**

To assess parental depressive symptoms the Beck Depression Inventory-II (BDI) was used. Beck Depression Inventory is the most frequently used screening instrument in research on depression. Beck Depression Inventory was first developed by A. T. Beck in 1961. The inventory was reevaluated and revised in 1978 as a second form. The BDI total score correlates significantly with diagnoses of clinical depression (9,10), and it has well-established psychometric properties in both psychiatric and non-psychiatric samples (11). A quadruple Likert-type scale consisting of 21 questions was used to measure the severity of symptoms associated with depression. Each question was scored between 0 and 3, and the total score ranged from 0 to 63. The cut-off score of the scale was determined as 17 in the Persian validity and reliability study. The scale was classified as follows: a score of 0-10 denoted no depression, a score of 11-17 denoted a mild level of depression, a score of 18-23 signified a moderate level of depression and a score of 24 and above denoted a severe level of depression. The Cronbach’s alpha validity coefficient was 0.90 for mothers. The BDI has had high internal consistency, with alpha coefficients of 0.86 and 0.81 for psychiatric and non-psychiatric populations, respectively (12). In this study, the alpha coefficients for patients were found as 0.85.

**Statistical analysis**

The data obtained was analysed using SPSS 16.0 (Statistical Package for Social Science for Windows) package program. Descriptive statistics of the mothers demographic characteristics (age, gender, education level, economic status, employment status, family type, place of residence, number of children, number of siblings etc.), and the results of the BDI scores were calculated were analysed using the Student’s t-test, the Mann-Whitney U test, and the One-Way Anova and Kruskal-Wallis variance analysis. A value of p<0.05 was considered statistically significant.

## Results

The mean age of the children in this study was 9.73 years (SD = 5.33), with a range of 1 to 12 years. The average time since diagnosis was 4.53 month (SD = 2.90), with a range of one month to 1 year. The mean age at the time of diagnosis was 5.2±5.2 years. Of the 58 children in the study, there were 39 males and 19 females. The average age of the mothers was 45.3 ±1.3 (range= 21-86 years). The mothers’ occupational levels consisted of 6.7% unemployed, 17.2% homemakers, 22.4% unskilled laborers, 11.2% semiskilled laborers, 19.4% skilled laborers, 7.5% professionals, and 14.9% students. Occupational level was unknown for 1 participant (0.7%). Education levels were varied with 5.2% having less than a high school diploma, 28.4% having a high school diploma, 44% having attended some college, 11.2% with an associate’s degree, and 11.1% with a bachelor’s degree or higher. The majority of the sample (99%) were married or living together; the mean length of the parental relationship was 8.4 years (SD = 4.4). Among the families, 65.9% were middle income, and 22.7% were low income.

A varying number and severity of symptoms of current depression were identified by mothers on the BDI, with a mean score of 11.31 (SD = 10.06), in the non-depressed range (Table I). Although, the mean fell within the non-depressed range, the distribution of the number and severity of symptoms women were reporting covered the range from non-depressed to severely depressed, with a large portion (70.6%) indicating symptoms in the severe range. Based on BDI normative standards provided in the BDI manual, 41 participants (70.6%) indicated their current depressive symptoms as being in the severe range (BDI score greater than 25), 12 participants (20.6%) indicated symptoms in the moderate range (BDI score between 14 and 24), and 5 participants (8.6%) indicated symptoms in the mild range or no depression (BDI score between 0 and 13).

As shown in Table II, the highest prevalence rate of depression (25.8%) is seen in the age group of 35 to 39 years and the lowest rate (17%) in the age group of 19 to 24 years (p=0.643). The highest prevalence rate of depression (62%) is seen in mothers with an income level of less than 500,000 Tomans monthly (p=0.05). The prevalence of depression then decreases as the income increases. The highest and lowest rates of depression were recorded in the mothers with female and male childs, respectively.

**Table I:** Distribution of mothers BDI scores

**BDI**	**Frequency**	**Percent**
**0-13 points**	5	8.6%
**14-24 points**	12	20.7%
**≥25 points**	41	70.7%
**total**	58	100%

**Table II:** Distribution of mothers’ depression levels by age, educational level, employment status, number of children and their gender

**Variable**	**No depression** ** (n=5)**		**Mild Depression ** **(n=12)**		**Severe Depression** **(n=41)**		**Total **		**P value**
	**Frequency**	**%**	**Frequency**	**%**	**Frequency**	**%**	**Frequency**	**%**	
**Age** **19-24** **25-29** **30-34** **35-39** **≥40**	1 1 1 2 0	20 20 20 40 0	3 2 3 2 2	25 16.7 25 16.7 16.6	5 7 8 11 10	12.2 17 41.5 26.8 24.4	9 10 12 15 12	17 17.2 20.6 25.8 20.6	0.643
**Education Level** **Illiterate** **Elementary or higher** **High school ** **University graduated**	2 1 1 1	40 20 20 20	4 3 3 2	33.3 25 25 16.7	4 19 11 7	9.7 46.3 26.8 17	10 23 15 10	17.2 39.6 25.8 17.2	0.05
**Employment Status** **House working (45)** **Working (13)**	4 1	80 20	9 3	75 25	32 10	78 22	45 13	77.5 22.5	0.431
**No. children** **1-2** **3-4** **>4**	1 2 2	20 40 40	3 6 3	25 50 25	13 22 6	31.7 53.6 14.7	17 30 11	29.3 51.7 19	0.642
**Gender of the child** **Female** **Male**	3 2	75 25	7 5	58.3 31.7	29 12	70.7 29.3	39 19	67.2 33.8	0.542
**Income (Monthly)** **0-5000(Tomans)** **5000-1000,000** **≥1000,000**	2 2 1	40 40 20	6 4 2	50 33.3 16.7	28 8 5	68.3 19.5 12.2	36 14 8	62 24.2 13.8	0.05

## Discussion

The diagnosis and subsequent treatment of childhood leukemia is undeniably stressful for any family. A parent’s ability to manage his or her distress during treatment of the child is vital as there may be potential impact on the well-being and long-term psychological adjustment of both parents and child. The multinomial predictive model demonstrated that baseline maternal age and education; child health-related quality of life; child cognitive problems; and family functioning, resources, and demands play an important role in determining membership in a specific depressive symptoms trajectory (13,14).

Various studies have shown that mothers display symptoms such as hopelessness, despair, anger, stress, anxiety, and depression (15). The mean total BDI score this study was higher when compared to the mean total BDI score amongst Turkey and Pakistan mothers of children with leukemia (15,16).

In this study, the prevalence of depression in mothers was as high as 91%. Mild depression was seen in 69% of mothers, moderate in 20.6%, severe and very severe in 70.6%. Ghufran et al in a study in Pakistan reported that the prevalence of depression in mothers was as high as 78%. Mild depression was seen in 69% of mothers, moderate in 25%, severe in 5% while 1% had very severe depression (8). In other study in Pakistan, Iqbal et al have reported more than 65% of mothers of children with leukemia were found to be depressed (16). Roddenberry et al have suggests that an increased symptom of depression in mothers is related to significantly lower ratings in quality of life for their children (17). In a consistent study with conducted in Turkey reported that 88% mothers were depressed. Mild depression was reported in 22.7 % and major depression in 61.5% (18).

In the present study, there was not a significant correlation between mother’s employment and their depression levels. Similarly, Erkan et al when were examined to determine whether the depression levels of mothers varied with respect to their employment, they have not found a meaningful difference was found between depression levels and mothers’ employment. Although, some research studies have reported parallel findings (18).

In the present study, it was a meaningful difference between depressions levels of mothers with respect to the income level of the family. While, Erkan et al when examined to determine whether the depression levels of mothers varied with respect to the income level of the family, they have not found statistically meaningful difference (18). Some other studies showed that income had no relation to mother’s psychological health, burning out, family functions or stress levels. Dunst et al. have reported that there was no meaningful relationship between people’s income and perception of financial resources (19). In yet another study, Pittman and Loyd (1988) showed that life satisfaction depended more on the adequacy of one’s financial resources and whether they were able to meet their needs than merely on their income level (20).

## Conclusion

There is importance of incorporating mothers into the treatment process during the diagnosis and treatment of their children with leukemia. This study concludes that a majority of attending mothers of children with leukemia suffers from severe depression and it is associated with some factors. This study results may be useful for health care professionals as part of the initial consultation when diagnosing childhood leukemia so as to prevent any potential negative impact of maternal depressive symptoms on child health outcomes. However, further well-designed study need to conduct on a large number of mothers or both parents in order to make any logical conclusions, and finding socioeconomic and related factors on mother’s depression level.
